# Patient freedom to choose a weight loss diet in the treatment of overweight and obesity: a randomized dietary intervention in type 2 diabetes and pre-diabetes

**DOI:** 10.1186/1479-5868-11-64

**Published:** 2014-05-16

**Authors:** Leah T Coles, Elly A Fletcher, Claire E Galbraith, Peter M Clifton

**Affiliations:** 1 From the Nutritional Interventions Lab, Baker IDI Heart & Diabetes Institute, 75 Commercial Rd, Melbourne, VIC 3000, Australia; 2 Baker IDI Heart &Diabetes Institute, 75 Commercial Rd, Melbourne, VIC 3004, Australia

**Keywords:** Diabetes mellitus, Type 2, Prediabetic state, Weight loss, Obesity, Choice, Preference

## Abstract

**Background:**

Offering the overweight or obese patient the option of choosing from a selection of weight loss diets has not been investigated in type 2 diabetes. The aim of the study was to investigate if the option to choose from, and interchange between a selection of diets (“Choice”), as opposed to being prescribed one set diet (“No Choice”), improves drop out rates and leads to improved weight loss and cardio-metabolic outcomes.

**Methods:**

The study was a 12 month, randomized parallel intervention. A total of 144 volunteers with type 2 diabetes or pre-diabetes and a BMI >27 were randomized to “No Choice” or “Choice”. Those in the No Choice group were placed on a set weight loss diet (CSIRO) with no change permitted. Those in the Choice group could choose from, and interchange between, the CSIRO, South Beach or Mediterranean diets.

**Results:**

There were no differences in attrition rates or weight loss between the “Choice” and “No Choice”. In a secondary analysis of the intention-to-treat weight loss data with last measured weight carried forward gave a highly significant diet group by time by gender interaction (p = 0.002) with men doing better in the No Choice group overall (maximum difference “No Choice “-2.9 ± 4.6 kg vs. “Choice”-6.2 kg ± 5.3 kg at 6 months) and women doing better in the Choice group overall (maximum difference Choice -3.1 ± 3.7 kg vs. “No Choice” -2.0 kg ± 2.6 kg at 6 months).

**Conclusions:**

Men prefer direction in their weight loss advice and do less well with choice. A gender-specific approach is recommended when prescribing weight loss diets.

**Trial registration:**

anzctr.org.au ACTRN12612000310864.

## Background

The diagnosis of obesity-associated type 2 diabetes can reduce a person’s life expectancy by 8-10 years [1] and quality of life, particularly as a result of complications [2,3]. However, morbidity and mortality related to diabetes can be attenuated by a 15 - 20% reduction in body weight [1]. It is also possible, through lifestyle interventions, to prevent or delay the development of diabetes in ‘at risk’ individuals with pre-diabetes [3].

Lack of adherence to the weight loss plan prescribed is one of the challenges when recommending dietary modifications to free-living individuals. In dietary interventions, compliance and motivation of the participant generally decrease over time, regardless of the dietary regimen prescribed [4,5]. Changing a dietary pattern when it is either failing or because of boredom may produce a better result than forcing the participant to persist with the same pattern or drop out. Behavioural choice theory suggests that patients who receive the treatment that they prefer will have better outcomes in the context of a behavioural modification [6]. In chronic conditions, and particularly with regard to dietary modifications, the involvement and preferences of the patient may be particularly important as treatments are likely to require considerable long term lifestyle changes. Normal clinical practice is rarely as flexible as this with few practitioners endeavouring to determine patient needs and likes but we believe this is essential for long term success. Few lifestyle intervention studies have investigated choice or preference of treatment in terms of obesity and they have mostly been short in duration or small in size. In a small pilot study, Murray [7] found no differences between the group who received their preference and those who did not. Similarly, Renjilian found no difference comparing those who received their preferred treatment (group or individual therapy) for obesity and group therapy was far more successful [8]. It is possible in this study that the power of group therapy trumps the loss of choice. In contrast to the behavioural choice theory, Burke *et al*. in a large 18 month study found that those who received their preference had less weight loss than those who did not [9]. A larger two year study by Borridale *et al.*[10] found that those who did not receive their preference did significantly better than those who did. No gender effects were explored. Thus overall the literature would suggest that choice has either no effect or counterintuitively leads to worse outcomes in obesity trials. This may relate to the reasons people have become obese in which free choice is clearly detrimental.

No dietary preference weight loss studies have been performed dealing specifically with diabetes or pre–diabetes. To our knowledge there have also been no dietary intervention studies investigating preference of treatment that have allowed subjects to change diets during the intervention. Weight loss is greater in men than in women in most studies, including those in people with type 2 diabetes [11]. In the present work, we conducted a 12 month weight loss study in people with diabetes and pre-diabetes with the hypothesis that giving the participant the ability to choose from and interchange between a selection of diets, as opposed to being prescribed a set diet throughout, will (i) reduce dropout rates, and (ii) lead to improved weight loss and cardio-metabolic outcomes. We expected men would lose more weight than women and that weight loss would be greater with dietary choice in both groups.

## Methods

### Subjects

A total of 144 volunteers with a prior diagnosis of type 2 diabetes or pre-diabetes (self-reported as diagnosed by a physician) aged 40 – 75 y and a current BMI >27 kg/m^2^ were recruited, primarily using advertisements in a diabetes magazine and the daily newspaper. Potential participants were posted a brief summary of the principles of each of the three diets and were further able to view the diet plans during a pre-enrolment information session where they were told that all three diets had been shown to be effective in weight loss, with no bias towards any one diet. At the information session, participants gave consent and selected their diet of choice should they be randomly allocated to the Choice group. At the baseline visit they were given full dietary information for the CSIRO diet (No Choice group) or the diet that they had earlier selected. Informed written consent was obtained from all volunteers, and the study was approved by the Alfred Health Human Ethics Committee (24/11).

Subjects on any medication for type 2 diabetes, including insulin, were considered eligible. Exclusion criteria were prior gastric surgery for weight loss, already following a weight loss diet, taking appetite-altering drugs, or an unwillingness to adhere (e.g. they only wanted to follow one particular diet if enrolled in the study) to one or more of the experimental diets for 12 months (excluding short periods of up to three days). Subject characteristics are given in Table 1.

**Table 1:** **Baseline characteristics for all participants who enrolled in a 12 month weight loss trial**^**1**^

	**Diet group**		
	**All (*n*** **= 144)**	**No Choice (*n*** **= 73)**	**Choice (*n*** **= 71)**
Age (years)	58.3 ± 7.4	58.5 ± 6.9	58.0 ± 7.9
Weight (kg)	100.7 ± 17.6	100.6 ± 18.6	100.7 ± 16.7
BMI (kg/m^2^)	34.9 ± 5.4	35.0 ± 5.5	34.9 ± 4.8
HbA_1c_ (%)	7.1 ± 1.3	7.1 ± 1.2	7.2 ± 1.4
Pre-diabetes (*n*=)	24	11	13
Diabetes (*n*=)	120	62	58
Male (*n*=)	78	38	40
Female (*n*=)	66	35	31

^1^Data are means ± SD.

There was no significant difference between participants in the Choice versus the No Choice group for any variable or characteristic using one-way ANOVA.

HbA_1c_: glycosylated haemoglobin.

### Study plan

The study consisted of a 12 month parallel dietary intervention (May 2011 to October 2012). Subjects were randomized by random number generated using computer software (Excel 2007, Microsoft Corporation, Redmond, WA) to either the “No Choice” or “Choice” group after consenting to participate with an allocation ratio (Choice: No Choice) of 1 : 1.03. All enrolment procedures (random number generation, participant enrolment and assignment to one of the two groups) were undertaken by one or more of the researchers using an identical procedure to ensure consistency. Those allocated to the Choice group (*n* = 71) could choose which of the three study diets they wanted to follow and were able to seek permission from the research team to switch between these diets at any time. Those in the No Choice group (*n* = 73) were placed on the CSIRO diet without any option to change diets. Dietary advice was given by an Honours student and a Nutrition PhD. No dietetic professionals were used. Both knowledge transfer about the diet composition as well as motivation was explored in the diet sessions. Behavioural issues such as snacking and alcohol use which would be common to all diets were explored.

Primary outcomes were weight and HbA_1c_. Secondary outcomes were fasting glucose, triglycerides, cholesterol (total, HDL and LDL) and high sensitivity c-reactive protein (hs-CRP) measured at baseline, 3 months, 6 months and 12 months using venous fasting blood samples. Weight (Model: Well-being, A&D Company Limited, Tokyo, Japan), blood pressure (BP) (OMRON Automatic Blood Pressure Monitor, Model HEM-907, OMRON Corporation, Kyoto, Japan) and height (using a stadiometer) were recorded at baseline. In between the baseline and 3 month visits, participants met with a member of the research team fortnightly, with only weight and BP recorded. At these fortnightly visits, further dietary advice was provided to all participants during a one-on-one 20 minute consultation. Participants were encouraged by the research team to return to the clinic to be measured regardless of whether they complied with any dietary protocol. For the remainder of the study (excluding the 3, 6 and 12 month visits), visits increased to 6 weeks apart and changed to researcher-led group sessions of an hour in duration covering nutrition education (e.g. interpreting nutrition information on food labels) and behavioral issues with regard to weight loss and weight maintenance. There was no segregation of participants by diet group allocation (‘No Choice’ or ‘Choice’) or diet (CSIRO, SB, MED) for these group sessions. All data was collected onsite at Baker IDI Heart & Diabetes Institute, Melbourne, Australia.

#### Physical activity

At the commencement of the study, participants were provided with a basic pendulum pedometer (Be Active Step by Step, Model WWA2026, Pedometers Australia, Cannington, WA, Australia) and log book to record daily step count as motivation to follow the research team’s recommendation of least 30 min of physical activity (PA) most days [12]. A self-report questionnaire about PA patterns (type of PA, frequency and duration) was completed by participants at baseline and 12 months.

#### Diets

The three experimental diets were designated: South Beach (SB) [13], Mediterranean (MED) and The CSIRO Total Wellbeing Diet (CSIRO) [14]. Main features of the three interventional diets are provided as supplementary information for comparative purposes (Additional file 1). When followed as prescribed, the CSIRO and MED diets provided approximately 6000 kJ/day for both men and women, whilst the SB diet was *ad libitum*.

The SB and CSIRO diets were prescribed as published [13,14], whilst the MED diet was based on that described by Shai *et al*. [15] with the following guidelines: 3 servings of fruit and 4 servings of vegetables daily, 2 servings each of olive oil (1 serving = 1 teaspoon), whole grains and dairy daily, 3 servings of nuts and 2 servings of legumes weekly, optional daily intake of red wine with meals (1 × 80 ml glass for women and 1 - 2 for men), and red meat no more than once per week, with poultry (2 - 4 servings weekly) and fish/shellfish (2 servings weekly) being the main sources of protein. Participants were given documentation explaining their diet plan including appropriate recipes. Participants in the ‘No Choice’ group were only given information on the CSIRO diet and similarly, ‘Choice’ participants were only given information on their selected diet.

There was no expectation from previous trials that there would be any difference in weight loss between the diets as low carbohydrate, low fat and Mediterranean style diets have all been shown to be equally as effective in terms of weight loss over two years [15]. Our primary hypothesis was that Choice (regardless of actual diet chosen) would lead to better outcomes than No Choice.

### Chemical analyses

Venous blood samples were analyzed for glucose, glycosylated haemoglobin (HbA_1c_), cholesterol (total, HDL, LDL), total triglycerides and hs-CRP. Glucose (hexokinase method), cholesterol (enzymatic method), triglycerides (glycerol phosphate oxidase method) and hs-CRP (immunoturbidimetric method) were analyzed on an Archicentre ci16200 (Abbott Laboratories, Abbott Park, IL). HbA_1c_ (Boronate Affinity HPLC method) was analyzed on a Primus CLC-385 (Primus Diagnostics, Kansas City, MO).

### Statistical analysis

A power analysis indicated 70 participants would be needed (35 per group completing) to provide 80% power to detect a 1.5 kg difference between Choice and No Choice at a significance level of 0.05 with a standard deviation of 2.2 kg. To account for withdrawals and loss to follow-up over 12 months the aim was to recruit at least 120 participants. The standard deviation was based on our own published weight loss studies. The difference of 1.5 kg chosen was based on the difference seen between the treatment incongruent group and the no strong preference group from Borradaile et al (10)”.

Repeated measures ANOVA was performed on weight at baseline, 3, 6 and 12 months (SPPS version 19.0, IBM Corp., Armonk, NY) on an ‘intention-to-treat’ (ITT) basis using last measured weight carried forward for non-completers and in a second analysis with the addition of 1 kg increase per 12 months, with ‘Choice’ or ‘No Choice’ as the primary dietary factor and gender as a second factor. Secondary analyses were change of diet (yes/no) and diabetes versus pre-diabetes. Baseline characteristics of the subjects in Choice and No Choice were compared using one-way ANOVA. Changes in baseline cardio-metabolic measures at 12 months for completers were analyzed using repeated measures ANOVA. Retention rates, difference in physical activity levels and group session attendance rates between diet groups were also assessed by Chi-squared analysis. Medication changes (none, decreased or increased) were analyzed by univariate ANOVA. Correlations between group session attendance and weight loss were determined using a Pearson’s Correlation. A value of p < 0.05 was accepted as significant in all cases. Data are shown as mean ± SD, error bars are ± SE.

## Results

**Figure 1:**
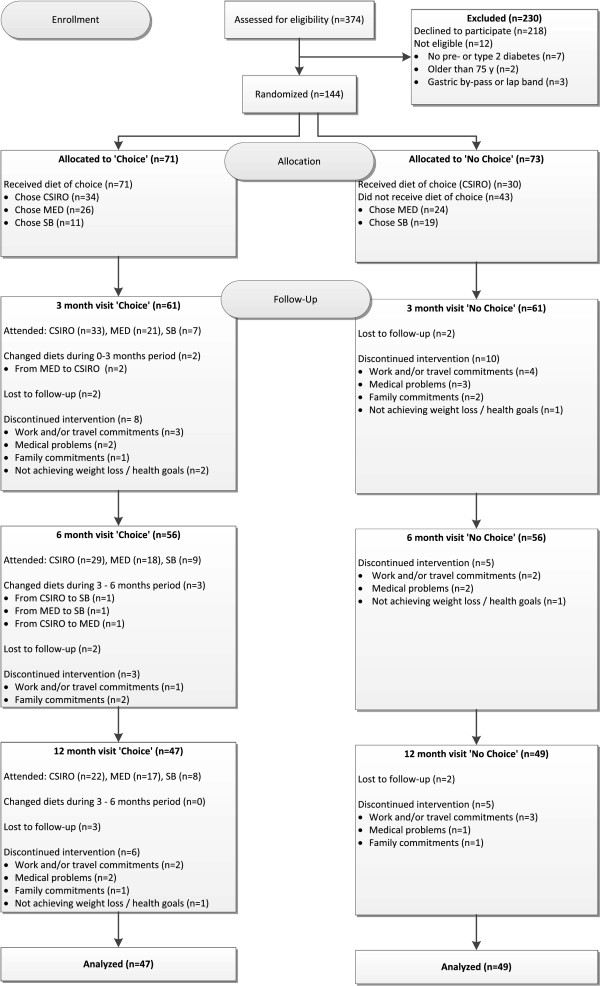
CONSORT-style flow chart.

Baseline characteristics of all subjects (*n* = 144) are given in Table 1. There was no significant difference between participants in the Choice versus No Choice group in terms of baseline age, weight, HbA_1c_ or BMI for these participants, nor for the cohort (*n* = 96) that completed the entire trial (data not shown). A CONSORT-style flow chart detailing numbers of participants at enrollment, allocation, follow-up (3, 6 and 12 months) and analysis is shown (Figure 1).

### Weight changes at 12 months

**Figure 2:**
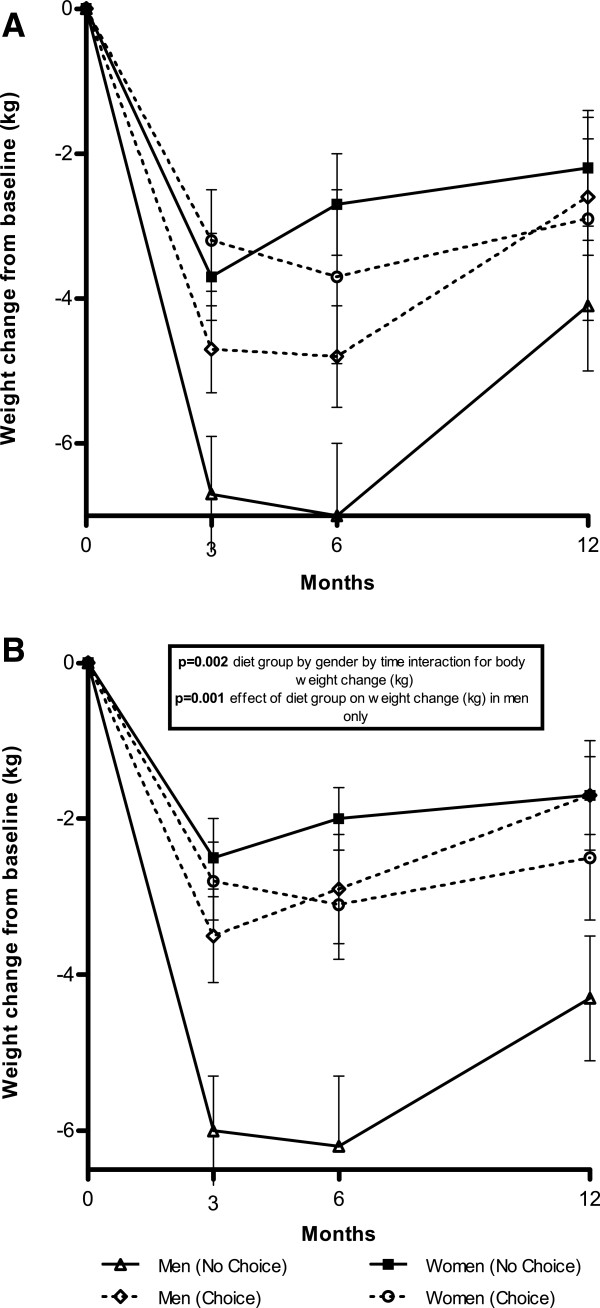
**Weight change (kg) from baseline by diet group and gender. A**. Shows only those participants who completed the 12 month weight loss program. **B**. Shows weights for all participants enrolled at baseline with weights carried forward from last known measurement for participants who discontinued the weight loss program. Data are means, error bars represent SE. P-values determined using repeated measures ANOVA. Participant numbers were: **A**. No Choice – women (n = 21) and men (n = 28); Choice - women (n = 18) and men (n = 29); and **B**. No Choice – women (n = 35) and men (n = 38); Choice women (n = 31) and men (n = 40).

There was no significant difference between weight loss for the 12 month completers in the No Choice (-3.5 ± 4.5 kg or -3.4 ± 4.5%) and Choice (-2.7 ± 5.0 kg or -2.8 ± 4.8%) arms over time. Weight change (kg) as categorized by diet group (Choice or No Choice) and gender over time is given both for 12 month completers (Figure 2A) and all enrolled participants on an ITT basis (Figure 2B). There was no significant effect of gender on weight loss in either the completers or the full cohort (ITT). Analysis of the ITT weight change (kg) data gave a highly significant diet group by time by gender interaction (p = 0.002) with men doing better in the No Choice group (maximal difference -2.9 ± 4.6 kg vs. -6.2 kg ± 5.3 kg at 6 months) and women doing better in the Choice group (maximal difference-3.1 ± 3.7 kg vs. -2.0 kg ± 2.6 kg at 6 months). In men alone, there was a significant effect of diet group allocation (Choice or No Choice) over time on absolute weight loss (p = 0.001). Even with the same diet (CSIRO) men on No Choice did better (weight loss of 4.6 kg versus 2.4 kg for those in Choice), although this was not statistically significant (p = 0.17) because of the small sample size. There were no differences in women alone. After removal of the SB diet from these two analyses (n = 135 in the analysis), there remained a significant diet by time by gender interaction on weight loss (p < 0.05) and a significant effect (p = 0.007) in men alone on weight loss.

In another analysis in which 1 kg per year weight gain (or fractions thereof) were added to the weight loss of the dropouts the diet group by gender by time remained significant (p = 0.02). In men alone the difference was still significant overall with an adjusted 12 m weight loss difference of 4.1 kg in No Choice vs 1.5 kg Choice (p = 0.02).

If you analyse without those in the No choice group who got their desired CSIRO diet which removes 31 individuals there was still a diet by time by gender interaction (p = 0.03). If you remove those who didn’t get their preferred diet which removes 41 individuals there was still a diet by time by gender interaction (p = 0.02).

### Diet selection

Of the 71 participants who were allocated to the Choice group, 34 chose CSIRO, 26 chose MED and 11 chose SB at baseline (Figure 1). Due to the fact that participants were asked to select their diet preference before they were allocated to the Choice or No Choice group, it was possible to determine how many participants received their diet preference and also determine if there was a gender preference in the overall group. Of the people who were placed in the No Choice group, 24 had selected MED and 19 had selected SB as their diet preference. As such, just under half (41%) of the 73 participants in the No Choice group were placed on their diet of preference (CSIRO), compared to 48% of participants in the Choice group who chose CSIRO diet and would therefore have been given their diet of preference even if they had instead been placed in the No Choice group. There was no significant difference between diet groups regarding those who chose CSIRO and those who did not. There were significant (p < 0.05) gender differences between diet preferences (prior to diet group allocation) when comparing the proportion of males and females who selected SB versus MED, SB versus CSIRO and CSIRO versus MED. Men favored SB (9 f, 21 m) and MED (22 f, 29 m) whilst women favored CSIRO (35 f, 28 m). These gender ratios were similar to those for participants who were actually placed in the Choice group, i.e. women favored the CSIRO diet (19 f, 16 m), whilst the MED diet (9 f, 15 m) and SB diet (2 f, 9 m) were more popular amongst men, although differences in gender proportions were only significant for SB relative to the two other diets (p < 0.05).

### Diet changes

Only five participants in the Choice group changed diets during the study: two from MED to CSIRO, one from MED to SB, one from CSIRO to SB and one from CSIRO to MED (Figure 1). All diet changes occurred between 8 – 20 weeks and in all but one case, only women opted to change diets. Study staff were not aware of any participant in the No Choice group attempting to follow any diet plan other than CSIRO (i.e. had changed diets) nor any participant in the Choice group who had changed to another Choice diet without the prior knowledge of study staff. This was based on frequent questioning of participants by study staff during check-ups.

### Retention rates

Of the 144 participants at baseline, those remaining in the trial at key milestones were: 122 participants (85%) at 3 months, 112 participants (78%) at 6 months and 96 participants (67%) at 12 months (Figure 1). Retention rates (at 12 months) were similar for subjects with pre-diabetes (67%) and type 2 diabetes (68%). Although a larger number of men (73%) compared to women (59%) completed the trial, the gender difference was not significant. There was no instance of participant withdrawal due to a participant being randomly allocated to Choice (when No Choice was preferred) or No Choice (when Choice was preferred). Retention rates by allocated group (Choice and No Choice) were comparable (Choice: 66%, No choice: 67% at 12 months) with no significant difference between Choice and No Choice at 3, 6 or 12 months (p > 0.05). The withdrawal rate from the SB diet was significantly higher than for the other diets at 3 months (p < 0.05) with 4 out of the 11 subjects withdrawing in the first 12 weeks. Of the five withdrawals from SB, three were due to the participant not achieving their weight loss or health goals, which was not a common reason for withdrawal from the other diets (only two other participants gave this as a reason for withdrawal - both in the No Choice group). The most common reason for non-completion, generally, was due to work and/or travel commitments, making it difficult for participants to attend appointments during the study and/or to follow the prescribed diet. Because of the difficulties with the SB diet we have done a separate analysis excluding participants who chose this diet.

### Physical activity changes

Of the 96 completers, complete baseline and 12 month self-report data on physical activity (PA) was provided by 78 subjects (Choice n = 38, No Choice n = 40). For these 78 participants, an assessment was made of whether the participant was meeting the recommended [12] 150 min of physical activity per week at baseline and/or 12 months. At baseline, 52 participants (67%) were meeting PA guidelines, with no significant difference between the proportion in the Choice (68%) versus No Choice group (65%). At 12 months, 49 participants (65%) were meeting PA guidelines, with no significant difference between proportions in the Choice (61%) versus No Choice group (65%).

### Medication changes

A total of 19 participants (11 ‘Choice’ and 8 ‘No Choice’) had changes to diabetes medication (increase or decrease) during the study at the instruction of the participant’s physician (or the physician within the research team, who was blinded to the participant’s diet group, on a case by case basis as required). Approximately half of these participants (ten in total) were taking only oral diabetes medication, whilst the remainder were taking insulin exclusively or in combination with oral diabetes medication. With the exception of five participants (3 ‘No Choice’), all changes were a decrease in diabetes medication. Multiple diabetes medication reductions occurred for five participants, whilst four participants ceased all diabetes medication during the study, including one participant who was taking insulin at baseline. Change in body weight at 12 months (relative to baseline) was borderline significant (p = 0.07) when comparing subjects who had no diabetes medication changes (-2.4 kg weight change), diabetes medication reductions (-4.4 kg) or increases (1.8 kg).

### Group session attendance

Attendance rates at the four group dietary counselling sessions for the participants completing the 12 month program varied from 25% (one session) to 100% (four sessions) (Additional file 2). In both the Choice and No Choice groups, 100% attendance was evident in 40% of the group. There was no effect of group session attendance on weight loss for the 12 month completers when this was added as a covariate in the repeated measures ANOVA. However, there was a correlation between group session attendance and weight change (r = 0.21, p = 0.038).

### Cardio-metabolic changes

Changes in HbA_1c_ (%), fasting glucose, triglycerides, cholesterol (total, LDL and HDL) and hs-CRP at 12 months relative to baseline are given in Table 2. Importantly, there was no significant difference for participants in the Choice group relative to those in the No Choice group at 12 months. However, there was an overall effect of time on fasting glucose (p = 0.02), diastolic (DBP) (p = 0.02) and systolic BP (SBP) (p < 0.001).

**Table 2:** **Cardio-metabolic changes from baseline to 12 months for participants completing a 12 month weight-loss trial**^**1**^

**Variable**	**Diet group**					
	**All (*n*** **= 96)**		**No Choice (*n*** **= 49)**		**Choice (*n*** **= 47)**	
	**Baseline change**		**Baseline change**		**Baseline change**	
SBP (mmHg)	140.8 ± 18.1	-6.4 ± 17.4^§^	142.9 ± 19.5	-6.7 ± 17.5	138.5 ± 16.6	- 6.2 ± 17.4
DBP (mmHg)	84 ± 10.8	-3.9 ± 10.8^§^	85.0 ± 11.4	-5.0 ± 10.1	82.7 ± 10.2	-2.7 ± 11.6
HbA_1c_ (%)	7.02 ± 1.26	-0.15 ± 0.79	7.04 ± 1.28	-0.19 ± 0.82	6.99 ± 1.26	-0.11 ± 0.77
Fasting Glucose (mmol/L)	7.47 ± 2.28	-0.42 ± 1.75^§^	7.51 ± 2.50	-0.53 ± 1.94	7.41 ± 1.93	-0.31 ± 1.53
Triglycerides (mmol/L)	1.79 ± 0.89	-0.09 ± 0.89	1.85 ± 0.97	-0.26 ± 0.76	1.73 ± 0.79	0.09 ± 0.98
Total cholesterol (mmol/L)	4.50 ± 1.13	-0.01 ± 0.61	4.33 ± 0.94	-0.05 ± 0.60	4.67 ± 1.29	0.03 ± 0.63
HDL cholesterol (mmol/L)	1.20 ± 0.45	-0.05 ± 0.35	1.13 ± 0.24	-0.01 ± 0.16	1.28 ± 0.59	-0.09 ± 0.47
LDL cholesterol (mmol/L)	2.46 ± 1.01	0.12 ± 0.61	2.26 ± 0.89	0.15 ± 0.68	2.66 ± 1.10	0.08 ± 0.52
hs-CRP (mg/L)	2.96 ± 2.45	-0.36 ± 1.84	3.15 ± 2.72	-0.51 ± 2.13	2.76 ± 2.14	-0.21 ± 1.49

^1^Data are means ± SD, shown only for those participants who completed the 12 month trial.

^§^There was no effect of diet group for any variable (repeated measures ANOVA), and no significant difference between baseline measures and those at 12 months for any variable for any diet group (All, No Choice or Choice) except for change in overall SBP (p < 0.001), DBP (p = 0.02) and fasting glucose (p = 0.02) (repeated measures ANOVA).

DBP: diastolic blood pressure, HbA_1c_: glycosylated haemoglobin, HDL: high-density lipoprotein, hs-CRP: high-sensitivity c-reactive protein, LDL: low-density lipoprotein, SBP: systolic blood pressure.

## Discussion

Lifestyle interventions frequently require considerable effort and long-term commitment by the individual and their effectiveness is often hindered by lack of adherence. The present study investigated whether patient freedom to choose a weight loss diet and later change to a different diet (if desired) would lead to increased weight loss and/or improvements in HbA_1c_ .

### Participants in the choice group rarely changed diets

The fact that only five participants in the Choice group elected to change diets was an unexpected outcome and an important finding given the ability to change diets during a weight loss intervention has not been investigated previously. Instead of changing diets, participants not achieving their weight loss or health goals preferred to withdraw entirely from the study. This may have been because participants in the Choice group had already chosen the diet that they viewed as most likely to give them success or that participants in the Choice group had an inflated expectation of success compared to those in the No Choice group. The latter is consistent with other lifestyle intervention studies where participants who had the ability to select diets performed poorly compared to those who did not [10], [16]. In the present study, there was no significant difference in Choice and No Choice groups with respect to withdrawal rates or the proportion of participants in each group who received their diet of choice.

### Gender differences in diet preference, attrition rates and weight loss

Overall, males were well represented in the study and outnumbered the women. A key finding in the present study was the gender differences that existed with relation to dietary preference in terms of diet selection and weight loss outcomes.

#### Gender differences in initial diet preference

The strong male bias towards SB may have been due to a preference for animal protein amongst men, its simplicity (all foods were designated as ‘allowed’ or ‘not allowed’) or non-calorie restricted design. Men, however, coped poorly with this diet and either dropped out or had poor weight loss but even excluding this diet from the analysis showed that men did worse with choice. Women strongly preferred CSIRO. Other studies have shown that women are more likely to sacrifice their dietary regimen for their family’s food preference [17] and CSIRO was viewed by many participants as being ‘family friendly’. It is plausible that the women in our study, most likely responsible for the nutrition of the household, took into greater account the acceptability of the diet by other (particularly younger) family members than the men did.

#### Gender differences in familial support

A recent study by Mathew *et al*. [18] highlights the struggles with diet and nutrition among women in diabetes self-management. Other previous work is consistent in that women are supportive of their husband’s dietary changes and diabetes self-management, whilst men are less supportive of their wife in this regard [19,20]. Female participants more often complained about the lack of familial support and family pressures, including their family’s dislike at having to eat differently, which hindered their own compliance.

#### Gender differences in weight loss

The observed diet group (Choice or No Choice) by time by gender interaction suggests that both gender and its interaction with the option to choose or not choose between weight loss diets are important determinants of weight loss success. The results presented here support a gender-specific approach when offering nutritional advice for weight loss and diabetes management. Specifically, our findings suggest that men may achieve better weight loss outcomes when given a single option. Although our data suggest that offering an array of options to men for weight loss may not lead to the best outcome for the group, clearly within the group there would be many men who would welcome and do better with an array of choices. Some practitioners would also not be comfortable offering only one solution to men. The reasons for men to not do as well with a choice of diets and the ability to change diets is not clear but may relate to lack of confidence in decision making about food and a lack of knowledge compared with women. Dietary advice was given by an Honours student and a Nutrition PhD. No dietetic professionals were used. Both knowledge transfer about the diet composition as well as motivation was explored in the diet sessions. Behavioural issues such as snacking and alcohol use which would be common to all diets were explored even when those in the No Choice group who received their desired diet were removed from the analysis there was still a diet by time by gender interaction ie the loss of choice was the most important factor rather than the diet per se. Although in experimental tests of decision making with a variety of scenarios there are no differences between men and women [21] but with patients surveyed after an acute ischemic event men were reported to be more satisfied with the information received while women wanted more information although both agreed that only a minority of decision making was shared although this is what they both wanted. This suggests men were more content with the reality of following doctors decision [22]. In weight loss studies once a man has made a decision that weight loss is required (which they do much less often than women) they are much more successful at achieving weight loss regardless of the dietary intervention.

### Limitations of the present study

This study was limited by a lack of diet records, measures of satisfaction and a lack of alternative diets in the No Choice group to ensure no one in this group was given their desired diet. Underlying personal traits or behaviors related to the type of diet a person chose may have also effected their success in implementing behavioral lifestyle changes (as opposed to the actual diet plan). For example, people who enjoy cooking, previously shown to be a factor in successful weight management [23], may have chosen CSIRO whilst people who do not make good food choices may have preferred the ‘black and white’ rules of excluding carbohydrate in SB. Some individuals may have lost considerable weight regardless of the diet due to possessing a good support network, or the necessary skills and behaviors likely to lead to success. Despite the researchers’ best efforts to be impartial, there may have been bias in the nutritional counseling provided to one group (e.g. diet, gender, diet group) over another. Participants themselves may have had perceptions of what the best diet or diet group was and this may have influenced or been influenced by other participants during group sessions.

## Conclusions

In conclusion, the option to change dietary patterns did not improve retention rates in this weight loss intervention, although gender differences exist in terms of diet selection and weight loss outcomes. For men, clear direction was important. Current dietetic practice generally places little emphasis on gender-specific approaches when offering nutritional advice for weight loss and diabetes management. This is particularly pertinent in the case of women with chronic physical medical conditions who may be attempting to make lifestyle changes without their family’s support or willingness to adapt to different dietary patterns. Prescribing a set weight loss regimen to such a patient without consideration of such familial obstacles is more likely to be ineffective and may in fact contribute to the person’s low self-esteem (and further weight gain) from repeated failed weight loss attempts.

## Abbreviations

BP: Blood pressure; CSIRO: CSIRO diet; DBP: Diastolic blood pressure; HbA1c: Glycosylated haemoglobin; HDL: High-density lipoprotein; hs-CRP: high sensitivity c-reactive protein; LDL: low-density lipoprotein; MED: Mediterranean diet; PA: physical activity; SB: South Beach diet; SBP: Systolic blood pressure.

## Competing interests

The authors have no conflict of interest to report. PMC is co-author of the book “The CSIRO Total Wellbeing Diet”.

## Authors’ contributions

PMC, LTC, CEG and EAF designed research; CEG, EAF and LTC conducted research; PMC analysed data; CEG, LTC and PMC wrote the paper. PMC had primary responsibility for final content. All authors read and approved the final manuscript.

## Supplementary Material

Additional file 1 Main features of the three interventional diets. Click here for file

Additional file 2 Percentage of group sessions attended (%). Click here for file
