# Supplementary material for: Patient freedom to choose a weight loss diet in the treatment of overweight and obesity: a randomized dietary intervention in type 2 diabetes and pre-diabetes
Source: Int J Behav Nutr Phys Act. 2014 May 16;11:64. doi: 10.1186/1479-5868-11-64 (PMC4035903; doi:10.1186/1479-5868-11-64)
Supplement: Additional file 1 — Main features of the three interventional diets. [file 1479-5868-11-64-S1.pdf]

Additional File 1 - Main features of the three interventional diets

**Component**	**Diet**		
	CSIRO	MED	SB
Energy	kJ restricted (6000 kJ / day)	kJ restricted (6000 kJ / day)	*ad libitum*
Carbohydrate	**	***	*
Protein	***	**	***
Fat	*	***	***
Saturated fat	*	*	*

* indicates 'low'; ** indicates 'moderate'; *** indicates 'high'. Assignment of 'low', 'moderate' and 'high' are relative qualitative comparators between the three diets and not necessarily quantitative indications of amounts provided by the respective diet.

CSIRO, CSIRO Diet; MED, Mediterranean Diet; SB, South Beach Diet.
