# Supplementary figures and images for: Patient freedom to choose a weight loss diet in the treatment of overweight and obesity: a randomized dietary intervention in type 2 diabetes and pre-diabetes
Source: Int J Behav Nutr Phys Act. 2014 May 16;11:64. doi: 10.1186/1479-5868-11-64 (PMC4035903; doi:10.1186/1479-5868-11-64)

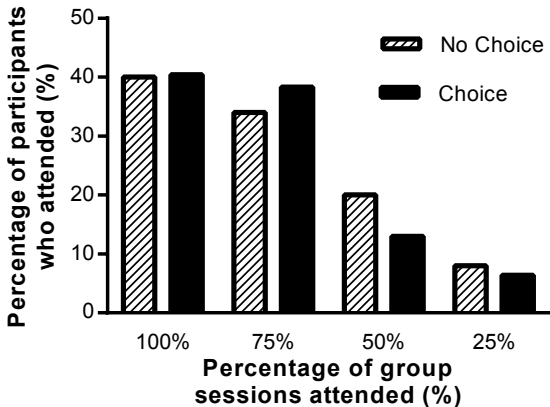

Supplement: Additional file 2 — Percentage of group sessions attended (%). [file 1479-5868-11-64-S2.pdf]
